# A double-blind, placebo-controlled comparator study of LY2140023 monohydrate in patients with schizophrenia

**DOI:** 10.1186/s12888-014-0351-3

**Published:** 2014-12-10

**Authors:** AnnCatherine M Downing, Bruce J Kinon, Brian A Millen, Lu Zhang, Lin Liu, Margarita A Morozova, Ronald Brenner, Tami Jo Rayle, Laura Nisenbaum, Fangyi Zhao, Juan Carlos Gomez

**Affiliations:** Eli Lilly and Company, Indianapolis, Indiana 46285 USA; Lundbeck LLC, Deerfield, IL 60015 USA; inVentiv Health Clinical, Burlington, Ontario L7L 6G4 Canada; Mental Health Research Center RAMS, Kashirskoye Shosse 34, Moscow, 115522 Russia; Neurobehavioral Research Inc, 74 Carmen Ave, Cedarhurst, NY 11516 USA

**Keywords:** Pomaglumetad methionil, Glutamate, Schizophrenia, Placebo, Risperidone, Pharmacology

## Abstract

**Background:**

Pomaglumetad methionil (LY2140023 monohydrate) is a potent and highly selective agonist for the metabotropic glutamate mGluR2 and mGluR3 receptors. We present results of a pivotal clinical study H8Y-MC-HBBM assessing the efficacy of LY2140023 in improving symptoms as a monotherapy in patients with an acute exacerbation of schizophrenia.

**Methods:**

Enrolled adult patients (ages 18–65) with schizophrenia who had experienced an exacerbation of symptoms within 2 weeks prior to study entry. Patients (N = 1013) were randomized 2:2:2:1 to treatment with placebo, LY40 mg twice daily (BID), LY80 mg BID, or risperidone (RIS) 2 mg BID for 6 weeks after a one-week blinded placebo lead-in. The primary outcome assessed change from baseline in the Positive and Negative Syndrome Scale (PANSS) total score in an overall schizophrenia population and a predefined subpopulation which excluded non-Hispanic white patients with the A/A genotype at the HTR2A SNP rs7330461.

**Results:**

Neither LY2140023 dose showed significant improvement compared to placebo on PANSS total in either population (1-sided p-value [significance level], overall: LY40, p = .154 [0.01]; LY80, p = .698 [0.01], subpopulation: LY40, p = .033 [0.0025]; LY80, p = .659 [0.0025], MMRM analysis). RIS statistically separated from placebo in both populations (p < .001 [0.05]). There were no statistically significant differences in the incidence of serious adverse events, and no seizures on LY2140023.

**Conclusion:**

LY2140023 treatment did not demonstrate efficacy in populations studied. Overall, LY2140023 treatment was generally well tolerated with no new adverse safety findings compared to previous trials. Further understanding of the role of glutamate as a therapeutic target in schizophrenia is needed.

**Clinical trials registration:**

A Phase 2, Multicenter, Double-Blind, Placebo-Controlled Comparator Study of 2 Doses of LY2140023 Versus Placebo in Patients With DSM-IV-TR Schizophrenia

ClinicalTrials.gov identifier: NCT01086748.

**Electronic supplementary material:**

The online version of this article (doi:10.1186/s12888-014-0351-3) contains supplementary material, which is available to authorized users.

## Background

Emerging evidence implicates dysregulation of the glutamatergic system as a key contributor to the symptoms of schizophrenia [1-4]. The mGluR2/3 receptors function as autoreceptors that, when stimulated by endogenous glutamate, are able to diminish the activity of hyperactive and dysregulated cortical pyramidal neurons [5]. Pomaglumetad methionil (LY2140023 monohydrate, hereafter referred to as LY2140023) is the methionine prodrug of the potent specific mGlu2/3 receptor agonist LY404039. It is anticipated that LY2140023 treatment may re-establish regulated and balanced glutamatergic cortical activity and improve psychosis.

An initial proof-of-concept study (Study HBBD) demonstrated that patients treated with LY2140023 (40 mg twice daily, BID) or olanzapine (15 mg) reported significant improvement versus placebo on positive and negative schizophrenia symptoms following 28 days of treatment [6]. However, the results of a second Phase 2 study (Study HBBI) were inconclusive. In Study HBBI, none of the LY2140023 dose arms (5 mg, 20 mg, 40 mg, 80 mg BID), nor the active control, olanzapine (15 mg), separated from placebo in the treatment of acute schizophrenia. Overall, while LY2140023 treatment was generally well-tolerated in both studies, in the HBBI study, seizures were reported in 4 patients treated with LY2140023 [7].

The primary objective of Study HBBM tested the hypothesis that LY2140023 (80 mg or 40 mg BID) would demonstrate significantly greater efficacy than placebo following 6 weeks of treatment, as measured by the change from baseline in the Positive and Negative Syndrome Scale (PANSS) total score in an overall schizophrenia population and/or in a predefined subpopulation which excluded non-Hispanic white patients with the A/A genotype at the serotonin 2A receptor (HTR2A) single nucleotide polymorphism (SNP), rs7330461. This predefined subpopulation was defined based upon pharmacogenetic analyses conducted from prior LY2140023 trials [8,9]. Secondary objectives focused on additional measures of efficacy and safety.

## Methods

### Study design

**Figure 1:**
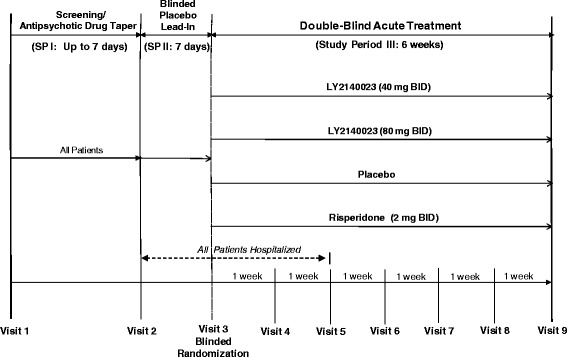
**The unblinded study design.** Study HBBM consisted of 3 periods: a screening phase, a 7-day placebo lead-in phase that was blinded to investigators and patients, and a 6-week randomized treatment phase. All patients were hospitalized from Visits 2–5. Patients could be discharged after that time based upon clinical presentation.

Study HBBM was a multicenter, randomized, double-blind, parallel, fixed-dose, Phase 2 study to assess the efficacy and safety of 2 dose levels of LY2140023 (80 mg or 40 mg BID) compared to placebo in patients with schizophrenia, with a risperidone treatment arm to assess assay sensitivity (Figure 1). The study consisted of 3 periods: a screening phase, a 7-day placebo lead-in phase that was blinded to investigators and patients, and a 6-week randomized treatment phase. All patients gave written informed consent prior to entering the study. The study protocol was approved by appropriate institutional review boards and conducted in accordance with the ethical principles stated in the Declaration of Helsinki.

#### Study period I (SP I)

Eligible patients were those with an accurate and reliable diagnosis of schizophrenia (based upon historical documentation and Structured Clinical Interview for DSM-IV Disorders [SCID] interview), who experienced an exacerbation of their illness 2 weeks prior to study entry (Visit 1), leading to a need for intensification of psychiatric care. Patients could be antipsychotic treatment naïve or have had prior exposure to antipsychotic medications and were not treatment refractory in the opinion of the investigator. Patients were excluded from study participation if they had any other current Axis I psychiatric diagnoses in addition to schizophrenia, a diagnosis of substance dependence or substance abuse, a history of one or more seizures, answered yes to any suicide-related behaviors within 1 month of Visit 1, participated in any clinical trial for which they received a study-related medication in the 6 months prior to Visit 1, were treatment refractory, or had demonstrated an inadequate response to treatment with risperidone, or for whom treatment with risperidone, LY2140023, or placebo was contraindicated.

An inclusion/exclusion review process was completed for each patient in the study prior to the patient being entered into the blinded placebo lead-in. All patients had to provide a blood sample for genotyping at screening to allow for appropriate classification within the predefined subpopulation.

#### Study period II (SP II)

Patients meeting enrollment criteria continued into a blinded (to patient and site) 7-day placebo lead-in treatment phase (SP II) from Visit 2 to Visit 3. A blinded placebo lead-in period was included in the study to improve signal detection by identifying patients whose change in PANSS total scores during this week suggested a high, anomalous placebo response. Patients were required to be hospitalized for a minimum of 3 weeks beginning at Visit 2.

#### Study period III (SP III)

A 6-week randomized double-blind treatment phase from Visit 3 to Visit 9 (Week 1–6) during which patients received one of the following study drugs administered orally BID: 40-mg LY2140023 tablets, 80-mg LY2140023 tablets, 2-mg risperidone over-encapsulated tablets, placebo tablets or capsules identical to LY2140023 and risperidone. Treatment with 80 mg BID LY2140023 was started at a dose of 40 mg BID for 3 days, followed by an increase to the target dose of 80 mg BID. Treatment with risperidone was started with 2 mg risperidone on the first day, and 4 mg (2 mg given BID) each day thereafter. The 2 doses of LY2140023 were selected based on previous clinical studies in humans. Dosing of risperidone was based on approved labeling.

### Assessments

The primary objective of this study tested the hypothesis that LY2140023, given orally to patients with schizophrenia at 80 mg or 40 mg BID, would demonstrate significantly greater efficacy than placebo following 6 weeks of treatment (Visit 9), as measured by the change from baseline in the PANSS total score [10], in one or more of the following populations: 1) overall schizophrenia population, 2) predefined subpopulation including all patients except non-Hispanic white patients (by self-report of race/ethnicity) who had the A/A genotype at the HTR2ASNP, rs7330461. Baseline was defined as the last non-missing observation at or before Visit 3.

To help reduce potential rating bias, improve reliability, and decrease variation, ratings were not performed by members of the treatment team but instead by blinded, independent raters. At the majority of sites (86%), centralized ratings via real-time video conference were conducted by an approved vendor (MedAvante, Hamilton, USA). In those countries where this technology was not available, local, blinded raters who were not a part of the treatment team performed the efficacy assessment. In both cases, the raters were blind to the study design, entrance criteria, and patient treatment assignment.

Key gated secondary objectives tested that LY2140023, at 80 mg or 40 mg BID, would demonstrate significantly greater improvement in functioning than placebo at Visit 9 in 1) the overall schizophrenia population, and 2) the predefined subpopulation, as assessed by the change from baseline in the Personal and Social Performance Scale (PSP) score [11].

Rates of discontinuation and time to discontinuation were compared between LY2140023 and placebo. The pharmacokinetics (PK) of LY2140023 and LY404039 were characterized.

Safety assessments included the incidence of treatment-emergent adverse events (TEAEs), neurological changes, vital signs, laboratory changes, and electrocardiogram (ECG) changes. Extrapyramidal symptoms (EPS) were assessed by the Barnes Akathisia Scale (BAS) for akathisia [12], Simpson-Angus Scale (SAS) for parkinsonism [13], and Abnormal Involuntary Movement Scale (AIMS) for dyskinetic symptoms [14].

Blood samples were collected from patients for genotyping (Verigene® mGlu Nucleic Acid Test; Nanosphere, Chicago, IL).

### Statistical methods

It was planned to randomize approximately 880 patients in a 2:2:2:1 ratio to LY2140023 (40 or 80 mg BID), placebo or 4 mg risperidone, respectively. This sample size provides a greater than 94% chance of at least one significant result from the four LY2140023 versus placebo tests (2 populations at 2 dose levels) associated with the primary objective. The power calculations were based on Monte Carlo simulations assuming an effect size of 0.3 and 0.4 in the overall population and the predefined subpopulation, respectively, and the predefined subpopulation makes up approximately 70% of the overall population.

The analyses were conducted on an intent-to-treat (ITT) basis that included all patients according to the treatment group to which they were assigned and received at least 1 dose. To increase signal detection, “placebo responders” who had a 25% or greater improvement in the PANSS total score (based on a 1–7 scale for each PANSS item) during the placebo lead-in period were excluded from the efficacy analyses. The resulting analysis population was referred to as the efficacy-evaluable ITT patient population.

To control the family-wise error rate associated with testing the alternate co-primary hypotheses, a fallback testing procedure [15] was employed. This procedure began by testing the efficacy of LY2140023 80 mg BID treatment first in the overall population, followed by testing the efficacy of LY2140023 40 mg BID treatment, and then conducting the corresponding tests in the predefined subpopulation [16].

For analysis of the PANSS total score in the overall population, the mixed model repeated measure (MMRM) included the fixed effects of treatment, pooled investigative site, visit, treatment-visit interaction, gender, predefined subpopulation (yes/no) as well as the continuous, fixed covariates of baseline score and baseline score-by-visit interaction. In the predefined subpopulation analysis, the categorical term of predefined subpopulation was excluded from the model. The within-patient errors were modeled using an unstructured covariance matrix. The Kenward-Roger approximation was used to estimate denominator degrees of freedom. The primary contrasts were the LY2140023 doses versus placebo comparisons at Visit 9.

The gated secondary outcome was change from baseline in the PSP score. Only when the null hypotheses from the primary testing sequence were rejected, would testing of the gated secondary hypotheses have commenced using a parametric gatekeeping procedure: test the efficacy of LY2140023 treatment versus placebo in the overall population, followed by testing in the predefined subpopulation.

The comparison between risperidone and placebo at Visit 9 was conducted at a 2-sided alpha level of 0.05 without adjustment for multiplicity.

Time to discontinuation due to adverse events (AEs), lack of efficacy, and for any reason were analyzed separately using Kaplan-Meier estimated survival curves; and the log-rank test was used to do comparisons.

The safety measures, including EPS scores, vital signs, body weight, body mass index (BMI), waist circumference, and ECG intervals, were analyzed using the MMRM analyses as specified previously. All the safety analyses were based on the ITT patients in the overall population. All tests of safety parameters were conducted at a 2-sided alpha level of 0.05 without adjustments for multiple comparisons.

For all other continuous variables such as patient’s age, height, baseline weight, BMI, waist circumference, baseline efficacy, and baseline EPS measures, a single-factor analysis of variance (ANOVA) with a fixed effect of treatment was used. For changes from baseline to last observed measure in laboratory analytes, the rank-transformed change from baseline data were used.

For all categorical data including patient’s gender, race, patient disposition, TEAEs, treatment-emergent abnormal, high, or low laboratory values, treatment-emergent EPS symptoms, Fisher’s exact test was used.

## Results

### Baseline and demographic characteristics

Among ITT patients in the overall population, there were no statistically significant imbalances among treatment groups with respect to baseline characteristics, psychopathology, or psychiatric history (Table 1). The overall population was comprised of 64.1% male and 35.9% female patients, with a mean age of 40.0 years. The mean duration of lifetime illness was 14.9 years, and the mean number of previous psychiatric hospitalizations was 7.8. Baseline EPS was similar across the treatment groups on the SAS total score and the BAS global score, although patients randomized to LY2140023 80-mg BID group had a higher baseline mean score on the AIMS total score than patients in the other treatment groups (p = .027).

**Table 1:** **Summary of patient baseline characteristics**

**Characteristics**	**PBO**	**LY40**	**LY80**	**RIS**	**TOTAL**	**Overall p-Value**
*ITT Overall Population*	N = 295	N = 292	N = 280	N = 142	N = 1009	
Age, years, mean (SD)	39.8 (11.4)	39.6 (11.5)	40.5 (11.5)	40.3 (11.1)	40.0 (11.5)	0.771
Gender, male, n (%)	181 (61.4)	196 (67.1)	183 (65.4)	87 (61.3)	647 (64.1)	0.422
Ethnicity, n (%)						0.612
Hispanic or Latino	13 (4.4)	11 (3.8)	15 (5.4)	9 (6.3)	48 (4.8)	
Not Hispanic or Latino	282 (95.6)	281 (96.2)	265 (94.6)	133 (93.7)	961 (95.2)	
Race, n (%)						0.899
American Indian or Alaska Native	1 (0.3)	0 (0.0)	1 (0.4)	0 (0.0)	2 (0.2)	
Asian	1 (0.3)	2 (0.7)	1 (0.4)	0 (0.0)	4 (0.4)	
Black or African American	102 (34.6)	100 (34.2)	94 (33.6)	47 (33.1)	343 (34.0)	
Multiple	6 (2.0)	1 (0.3)	6 (2.1)	1 (0.7)	14 (1.4)	
Native Hawaiian or Other Pacific Islander	2 (0.7)	1 (0.3)	1 (0.4)	1 (0.7)	5 (0.5)	
White	183 (62.0)	188 (64.4)	177 (63.2)	93 (65.5)	641 (63.6)	
Weight (kg), mean (SD)	81.4 (20.8)	81.4 (20.4)	83.3 (19.2)	82.7 (21.9)	82.1 (20.4)	0.622
Duration of Lifetime Illness , years, mean (SD)	14.5 (10.7)	14.6 (10.7)	15.6 (11.0)	15.2 (10.4)	14.9 (10.7)	0.607
Number of Past Psychiatric Hospitalizations, mean (SD)	8.0 (9.4)	7.2 (7.3)	8.2 (8.8)	7.4 (5.7)	7.8 (8.2)	0.439
Simpson-Angus Total Score, mean (SD)	0.5 (1.4)	0.4 (1.2)	0.5 (1.9)	0.6 (1.7)	1.5 (1.5)	0.718
Barnes Akathisia Global Score, mean (SD)	0.1 (0.5)	0.1 (0.5)	0.1 (0.4)	0.1 (0.4)	0.1 (0.5)	0.799
AIMS 1–7 Total Score, mean (SD)	0.4 (1.2)	0.2 (1.1)	0.6 (2.0)	0.2 (1.0)	0.4 (1.4)	0.027
ᅟ						
*Efficacy-Evaluable ITT Population*	N = 267	N = 267	N = 253	N = 132	N = 919	
PANSS Total Score, mean (SD)	84.3 (14.8)	83.7 (14.0)	84.2 (14.5)	84.0 (16.2)	84.1 (14.7)	0.971

*Abbreviations*: *AIMS* Abnormal Involuntary Movement Scale, *ITT* intent-to-treat, *kg* kilogram, *n/N* number of patients, *PANSS* Positive and Negative Syndrome Scale, *SD* standard deviation.

Groups: PBO = placebo; LY40 = LY2140023 monohydrate 40 mg; LY80 = LY2140023 monohydrate 80 mg; RIS = risperidone.

Among efficacy-evaluable ITT patients in the overall population, the mean PANSS total score at baseline was 84.1 (SD = 14.7) (Table 1).

### Patient disposition

**Figure 2:**
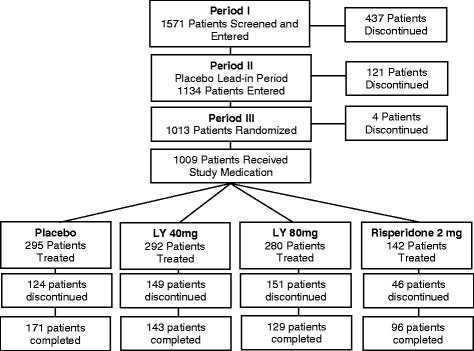
**Patient disposition.**

Of the 1571 patients who entered the study, 1134 patients entered the placebo lead-in period, 1013 were randomly assigned to treatment, 1009 received at least 1 dose of blinded study drug, 539 completed the study (Figure 2). Among all randomized patients, 90.7% (n = 919) were efficacy-evaluable ITT patients, 70.5% (n = 714) were ITT patients in the predefined subpopulation, and 64.5% (n = 653) were efficacy-evaluable ITT patients in the predefined subpopulation (Table 2).

**Table 2:** **Summary of study population for all entered patients**

**Analysis Population**	**PBO n (%)** ^**a**^	**LY40 n (%)** ^**a**^	**LY80 n (%)** ^**a**^	**RIS n (%)** ^**a**^	**Total n (%)** ^**a**^
Randomized Patients	N = 295	N = 293	N = 282	N = 143	N = 1013
*Completed*	171 (58.0)	143 (48.8)	129 (45.7)	96 (67.1)	539 (53.2)
ITT Patients in the Overall Population	295 (100.0)	292 (99.7)	280 (99.3)	142 (99.3)	1009 (99.6)
*Completed*	171 (58.0)	143 (48.8)	129 (45.7)	96 (67.1)	539 (53.2)
Efficacy-Evaluable ITT Patients in the Overall Population	267 (90.5)	267 (91.1)	253 (89.7)	132 (92.3)	919 (90.7)
*Completed*	154 (52.2)	136 (46.4)	116 (41.1)	90 (62.9)	496 (49.0)
ITT Patients in the Predefined Subpopulation	211 (71.5)	210 (71.7)	195 (69.1)	98 (68.5)	714 (70.5)
*Completed*	120 (40.7)	107 (36.5)	83 (29.4)	65 (45.5)	375 (37.0)
Efficacy-Evaluable ITT Patients in the Predefined Subpopulation	195 (66.1)	189 (64.5)	176 (62.4)	93 (65.0)	653 (64.5)
*Completed*	111 (37.6)	101 (34.5)	73 (25.9)	61 (42.7)	346 (34.2)

^a^Percentages were calculated based on all randomized patients per group.

*Abbreviations*: *ITT* intent-to-treat, *N/n* number of patients.

Groups: PBO = placebo; LY40 = LY2140023 monohydrate 40 mg; LY80 = LY2140023 monohydrate 80 mg; RIS = risperidone.

Among ITT patients in the overall population, the rate of study completion was significantly higher in the placebo group (58%) than in the LY2140023 40-mg BID (49.0%; p = .032) and 80-mg BID (46.1%; p = .005) groups (Table 3). The most common reasons for early discontinuation were perceived lack of efficacy–physician decision (159 patients, 15.8%), subject decision–consent withdrawn (107 patients, 10.6%), and AEs–physician decision (81 patients, 8.0%). The incidence of discontinuation due to perceived lack of efficacy-physician decision was significantly higher than placebo in the LY2140023 80-mg BID group (p = .025) and significantly lower than placebo in the risperidone group (p = .007). No other statistically significant differences were found.

**Table 3:** **Reasons for study discontinuation for all ITT patients in the overall population**

						**p-value**			
**Variable**	**PBO N = 295 n (%)**	**LY40 N = 292 n (%)**	**LY80 N = 280 n (%)**	**RIS N = 142 n (%)**	**Total N = 1009 n (%)**	**Overall**	**LY40 vs. PBO**	**LY80 vs. PBO**	**RIS vs. PBO**
**Completed**	171 (58.0)	143 (49.0)	129 (46.1)	96 (67.6)	539 (53.4)	<.001	0.032	0.005	0.059
**Discontinued**									
Adverse Event-Physician Decision	24 (8.1)	18 (6.2)	29 (10.4)	10 (7.0)	81 (8.0)	0.322	0.424	0.389	0.849
Adverse Event-Subject Decision	9 (3.1)	6 (2.1)	7 (2.5)	2 (1.4)	24 (2.4)	0.770	0.603	0.802	0.515
Entry Criteria Not Met	1 (0.3)	1 (0.3)	0 (0.0)	0 (0.0)	2 (0.2)	>.999	>.999	>.999	>.999
Lost to follow up	9 (3.1)	4 (1.4)	6 (2.1)	3 (2.1)	22 (2.2)	0.576	0.262	0.604	0.759
Perceived Lack of Efficacy-Physician Decision	39 (13.2)	56 (19.2)	57 (20.4)	7 (4.9)	159 (15.8)	<.001	0.057	0.025	0.007
Perceived Lack of Efficacy-Subject Decision	9 (3.1)	14 (4.8)	12 (4.3)	3 (2.1)	38 (3.8)	0.499	0.296	0.508	0.759
Protocol Violation	5 (1.7)	10 (3.4)	7 (2.5)	3 (2.1)	25 (2.5)	0.616	0.202	0.568	0.718
Scheduling Conflict	1 (0.3)	1 (0.3)	0 (0.0)	0(0.0)	2 (0.2)	>.999	>.999	>.999	>.999
Sponsor Decision	1 (0.3)	1 (0.3)	1 (0.4)	0 (0.0)	3 (0.3)	>.999	>.999	>.999	>.999
Subject Decision-Consent Withdrawn	26 (8.8)	33 (11.3)	30 (10.7)	18 (12.7)	107 (10.6)	0.596	0.339	0.483	0.235
Subject is Moving or has Moved	0 (0.0)	4 (1.4)	1 (0.4)	0 (0.0)	5 (0.5)	0.082	0.061	0.487	
Transportation Issues	0 (0.0)	1 (0.3)	1 (0.4)	0 (0.0)	2 (0.2)	0.668	0.497	0.487	

*Abbreviations*: *ITT* intent-to-treat, *N/n* number of patients.

Groups: PBO = placebo; LY40 = LY2140023 monohydrate 40 mg; LY80 = LY2140023 monohydrate 80 mg; RIS = risperidone.

Note: p-values are calculated from Fisher's exact test.

**Figure 3:**
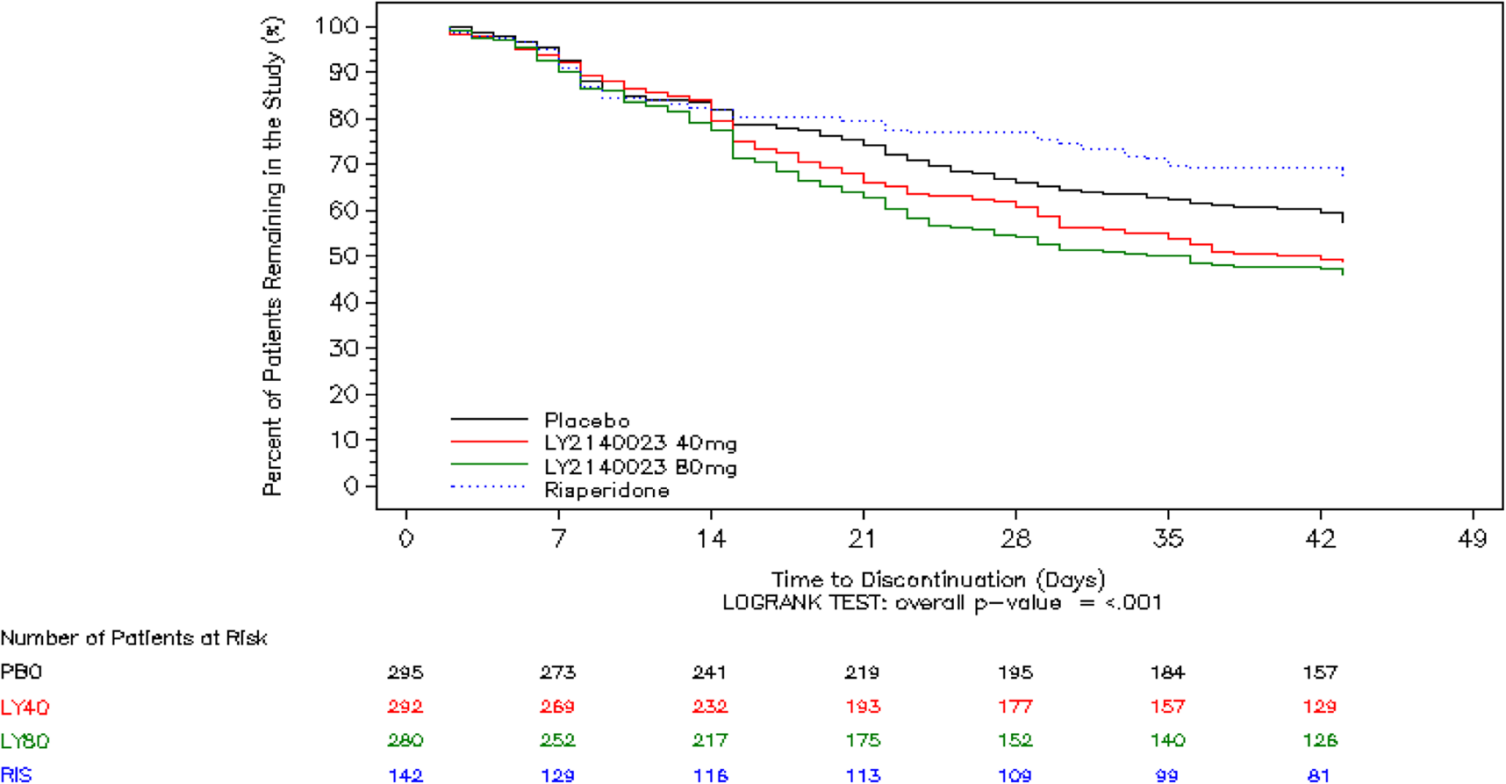
**Kaplan-Meier time to discontinuation for any reason; intent-to-treat patients in the overall population.**

There was a statistically significant overall difference among all treatment groups in time to discontinuation for any reason (p < .001) (Figure 3) and time to discontinuation due to lack of efficacy (p < .001) (data not shown). Compared to placebo, patients in the LY2140023 groups had a significantly shorter time to study discontinuation for any reason (LY2140023 40-mg BID group, p = .036; 80-mg BID group, p = .003) and a significantly shorter time to discontinuation due to lack of efficacy (LY2140023 40-mg BID group, p = .020; 80-mg BID group, p = .006). Patients in the risperidone group had a significantly longer time to discontinuation due to lack of efficacy compared to patients in the placebo group (p = .010). There was no statistically significant difference among all treatment groups in the time to discontinuation due to AEs (p = .174).

### Efficacy measures

#### Primary objective

**Figure 4:**
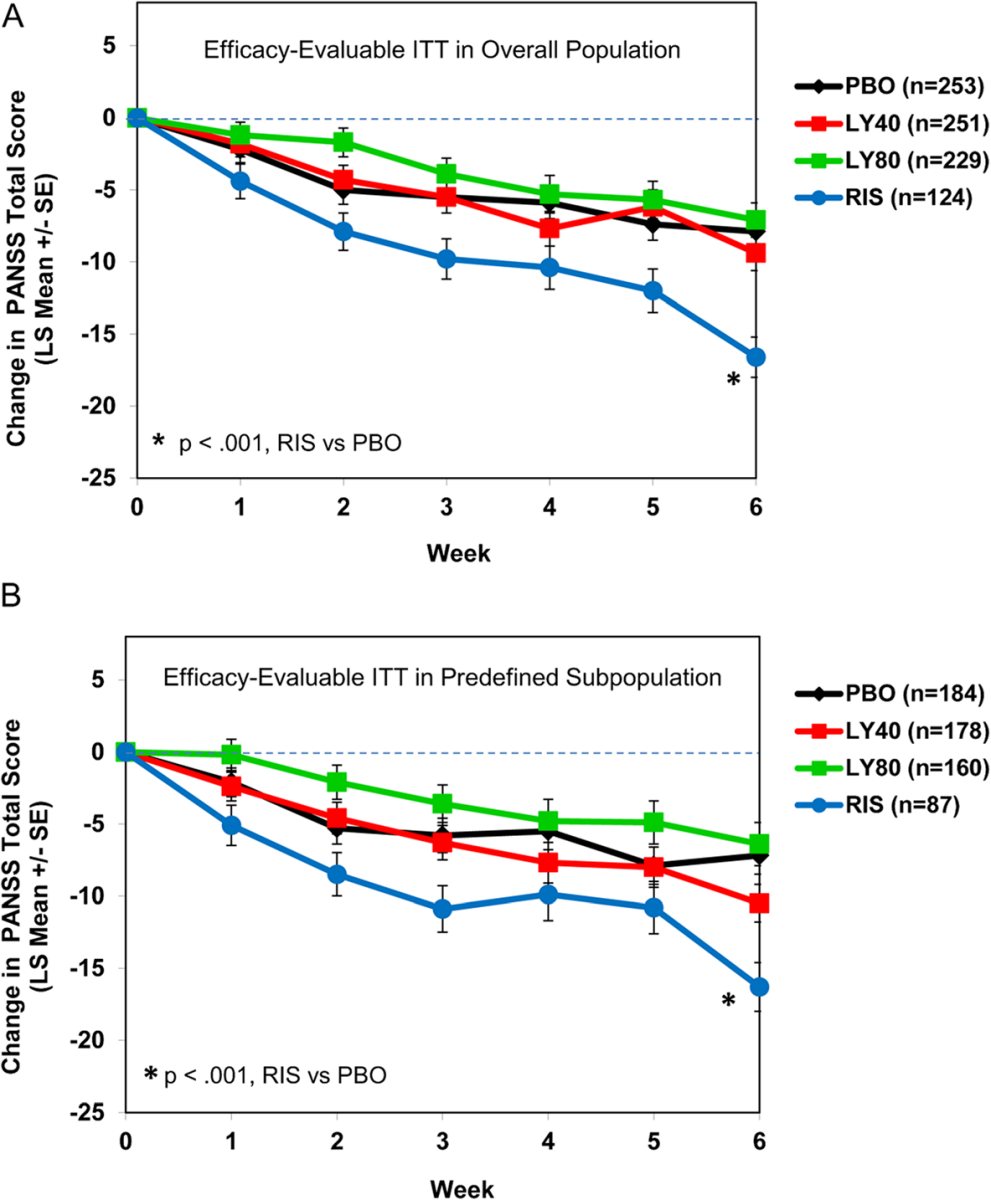
**Plot of weekly least-squares mean (+/−standard error [SE]) of efficacy measures by treatment.** Repeated measures analysis of change from baseline to each postbaseline visit (MMRM), efficacy-evaluable intent-to-treat patients in the overall population **(A)** and in the predefined subpopulation **(B)**. In the figures, Week 0 corresponds to study Visit 3, and Week 6 corresponds to study Visit 9.

Within-treatment-group improvement on PANSS total score from baseline was observed for all treatment groups at each visit for the efficacy-evaluable ITT overall population and the efficacy-evaluable ITT predefined subpopulation (Figure 4A,B). Following the testing procedure specified for the primary outcome, neither LY2140023 dose showed a statistically significant improvement on the PANSS total score at endpoint compared with placebo in either population (Table 4). Risperidone statistically separated from placebo on PANSS total score at endpoint in both populations (p < .001).

**Table 4:** **Primary analysis mixed-model repeated measures at visit 9 – PANSS total score in efficacy-evaluable ITT patients**

**Population**	**Treatment**	**N**	**LS Mean change**	**LS Mean change vs. placebo [1-Sided p-value]**	**Significance level (α, 1-sided)**	**Result of significance test (S/NS)**
**Overall population**	PBO	253	−7.9			
	RIS	124	−16.6	−8.7 [<.001]*	0.05*	Validity: S
	LY80	229	−7.1	0.8 [0.698]	0.01	Step 1: NS
	LY40	251	−9.4	−1.5 [0.154]	0.01	Step 2: NS
**Predefined subpopulation**	PBO	184	−7.2			
	RIS	87	−16.3	−9.1 [<.001]*	0.05*	Validity: S
	LY80	160	−6.4	0.8 [0.659]	0.0025	Step 1: NS
	LY40	178	−10.5	−3.3 [0.033]	0.0025	Step 2: NS

*Abbreviations*: *ITT* intent-to-treat, *LSMean* least-squares mean, *NS* not significant, *PANSS* Positive and Negative Syndrome Scale, *S* significant.

Groups: PBO = placebo; LY40 = LY2140023 monohydrate 40 mg; LY80 = LY2140023 monohydrate 80 mg; RIS = risperidone.

*For the risperidone group, the 2-sided p-value and significance level were used.

### Other efficacy measures

Since the relevant primary null hypotheses were not rejected, no testing of the gated secondary objectives was needed.

### Sensitivity analyses

For efficacy measures, all primary and secondary analyses were applied to the ITT overall population (included 90 placebo responders) and the ITT predefined subpopulation (included 61 placebo responders) to monitor sensitivity of the findings to the inclusion of placebo responders. Inclusion of the placebo responders did not change the results.

### Pharmacokinetic analyses and results

A parent-metabolite model was developed in which parameters for both LY2140023 and LY404039 were determined simultaneously. The HBBM data fit to models developed in previous studies of LY2140023 and was consistent with administration of oral LY2140023 (**HBBM** Additional file 1).

### Safety measures

A total of 44 (4.4%) patients experienced at least 1 serious adverse event (SAE) during the study. The most frequently occurring SAEs were schizophrenia-related events (preferred term: schizophrenia, n = 19, 1.9%; psychotic disorder, n = 5, 0.5%). All other SAEs were reported in <1% of patients. There were no significant between-group differences related to serious adverse events.

One patient in the LY2140023 80-mg BID group died secondary to cocaine intoxication. Two additional deaths occurred in patients who signed informed consent to participate but prior to receiving randomized treatment. One patient died of acute cardiac arrest during the screening period, and 1 patient committed suicide during the placebo lead-in period.

To ensure all potential seizure cases were appropriately confirmed as events, an external seizure adjudication committee was employed. Four cases were adjudicated with 3 being confirmed as seizures. Two patients experienced a seizure prior to randomization to study treatment. One patient who experienced a seizure was randomized to treatment with risperidone. No seizures occurred in patients taking LY2140023.

The overall incidence of AEs leading to study discontinuation was not significantly different between treatment groups (p = .208). Overall, 104 (10.3%) patients discontinued due to AEs. The percentage of patients who discontinued the study due to AEs were as follows: placebo, 11.2%; LY40, 7.9%; LY80, 12.9%; and risperidone, 8.5%. The most frequently reported AE leading to discontinuation in all treatment groups was schizophrenia (placebo, 4.4%; LY40, 2.7%; LY80, 4.3%;risperidone, 2.8%), followed by psychotic disorder (placebo, 1.4%; LY40, 1.0%; LY80, 1.4%; and risperidone, 0%). All other AEs leading to discontinuation occurred in <1% of patients overall.

As indicated in Table 5, there was a statistically significant overall difference observed among treatment groups in weight increase (with risperidone > placebo), restlessness, extrapyramidal disorder, dysmenorrhoea (with LY40 > placebo), sinusitis (with LY40 > placebo), asthenia, fatigue, and hyperhidrosis across all treatment groups.

**Table 5:** **Treatment-emergent adverse events preferred term by decreasing frequency ≥ 2% of patients or statistically significant comparison level ITT patients in the overall population**

						**p-value**			
**Preferred term**	**PBO N = 295 n (%)**	**LY40 N = 292 n (%)**	**LY80 N = 280 n (%)**	**RIS N = 142 n (%)**	**Total N = 1009 n (%)**	**Overall**	**LY40 vs. PBO**	**LY80 vs. PBO**	**RIS vs. PBO**
**Patients with > = 1 TEAE**	177 (60.0)	159 (54.5)	176 (62.9)	82 (57.7)	594 (58.9)	0.220	0.183	0.494	0.678
Headache	27 (9.2)	20 (6.8)	21 (7.5)	11 (7.7)	79 (7.8)	0.770	0.362	0.547	0.719
Schizophrenia	23 (7.8)	14 (4.8)	16 (5.7)	5 (3.5)	58 (5.7)	0.284	0.174	0.407	0.098
Nausea	14 (4.7)	22 (7.5)	16 (5.7)	5 (3.5)	57 (5.6)	0.338	0.172	0.708	0.627
Insomnia	21 (7.1)	10 (3.4)	15 (5.4)	10 (7.0)	56 (5.6)	0.188	0.064	0.396	>.999
Blood CK increased	15 (5.1)	12 (4.1)	17 (6.1)	4 (2.8)	48 (4.8)	0.485	0.694	0.717	0.327
Anxiety	9 (3.1)	11 (3.8)	12 (4.3)	5 (3.5)	37 (3.7)	0.887	0.657	0.508	0.778
Vomiting	6 (2.0)	11 (3.8)	13 (4.6)	5 (3.5)	35 (3.5)	0.350	0.229	0.102	0.347
Dyspepsia	5 (1.7)	7 (2.4)	11 (3.9)	8 (5.6)	31 (3.1)	0.105	0.575	0.130	0.033*
Constipation	8 (2.7)	7 (2.4)	7 (2.5)	5 (3.5)	27 (2.7)	0.900	>.999	>.999	0.765
Psychotic disorder	7 (2.4)	6 (2.1)	10 (3.6)	2 (1.4)	25 (2.5)	0.594	>.999	0.465	0.724
Agitation	3 (1.0)	8 (2.7)	10 (3.6)	1 (0.7)	22 (2.2)	0.102	0.141	0.050*	>.999
Weight increased	2 (0.7)	4 (1.4)	1 (0.4)	5 (3.5)	12 (1.2)	0.049*	0.449	>.999	0.039*
Restlessness	5 (1.7)	1 (0.3)	0 (0.0)	4 (2.8)	10 (1.0)	0.008*	0.216	0.062	0.481
Extrapyramidal disorder	3 (1.0)	0 (0.0)	6 (2.1)	0 (0.0)	9 (0.9)	0.029*	0.249	0.329	0.554
Dysmenorrhoea	0 (0.0	5 (5.2)	0 (0.0)	1 (1.8)	6 (1.7)	0.006*	0.019*		0.325
Sinusitis	0 (0.0)	5 (1.7)	0 (0.0)	1 (0.7)	6 (0.6)	0.013*	0.030*		0.325
Asthenia	2 (0.7)	0 (0.0)	0 (0.0)	4 (2.8)	6 (0.6)	0.002*	0.499	0.499	0.091
Fatigue	1 (0.3)	0 (0.0)	1 (0.4)	3 (2.1)	5 (0.5)	0.031*	>.999	>.999	0.103
Hyperhidrosis	0 (0.0)	0 (0.0)	1 (0.4)	2 (1.4)	3 (0.3)	0.019*		0.487	0.105

*Abbreviations*: *CK* creatine phosphokinase, *ITT* intent-to-treat, *N/n* number of patients, *TEAE* treatment-emergent adverse event.

Groups: PBO = placebo; LY40 = LY2140023 monohydrate 40 mg; LY80 = LY2140023 monohydrate 80 mg; RIS = risperidone.

Note: p-values are calculated from Fisher's exact test; * indicates nominal 2-sided p-value < = 0.05.

Analysis of change from baseline to endpoint in laboratory tests showed statistically significant changes within groups and when compared with placebo, although the majority of the changes are not viewed as clinically relevant. Mean eosinophil increases were statistically significant for both LY2140023 treatment groups in the overall comparison and pairwise versus placebo. At Visit 9, a statistically significant increase was seen for LY2140023 80-mg BID group. In the treatment-emergent abnormal analysis, both LY2140023 treatment groups showed a statistically significant increase in the number of patients displaying a high eosinophil count overall and versus placebo. However, no clinical correlates were noted with the eosinophil increases. Statistically significant increases in prolactin were noted for both LY2140023 80-mg BID (mean change, 1.16 μg/L) and risperidone (mean change, 50.90 μg/L) groups.

Statistically significant increases from baseline were seen on fasting glucose levels for patients treated with risperidone (p < 0.001) and placebo (p = 0.010). No statistically significant changes versus placebo in total cholesterol, HDL, LDL, or triglycerides were seen in the mean change laboratory reports. However, statistically significant reductions in total cholesterol from baseline were seen for both LY2140023 40 mg (p = .002) and LY2140023 80 mg (p < .001) treatment groups. Statistically significant reductions from baseline were also seen in triglycerides for LY2140023 80 mg treatment group (p = .028). All treatment arms showed statistically significant reductions in LDL cholesterol (placebo, p < .001; LY40, p = .018; LY80, p < .01; risperidone, p = .022); while no clinically relevant changes in HDL were noted across any of the treatment arms.

Overall, a significant difference was observed among treatment groups for potentially clinically significant (PCS) weight increases (p < .001), but not for PCS weight loss (p = .097). Compared with placebo, the risperidone group showed a PCS weight increase (14.6% of patients, p < .001) in the overall population. No significant differences were observed between placebo and either LY2140023 treatment group.

There were no clinically significant findings on vital signs or ECGs for patients treated with LY2140023 compared to placebo.

### Extrapyramidal symptoms

On the AIMS total score, the LY2140023 80-mg BID group had a statistically significant decrease compared to placebo at Visit 9 (MMRM analysis of change from baseline, p = .049). There were no statistically significant differences from placebo in any of the treatment groups for the BAS global score or SAS total score. There were no statistically significant differences from placebo in categorical analysis of treatment-emergent akathisia, parkinsonism, or dyskinesia in any of the treatment groups.

### Concomitant medications

There were no statistically significant differences between groups in the concomitant use of benzodiazepines/hypnotics/anxiolytics, anticholinergics, psychotropics, (antidepressants, mood stabilizers, and antipsychotics), antidiabetic and lipid/cholesterol lowering agents, or any other classes of medications during the study.

## Discussion

Study HBBM was a multicenter, randomized, double-blind, placebo- and comparator-controlled, parallel, fixed-dose, Phase 2 study conducted to assess the efficacy and safety of 2 dose levels of LY2140023 (80 mg BID or 40 mg BID) in patients with schizophrenia over 6 weeks of randomized treatment. Two populations, both the overall schizophrenia population and a predefined subpopulation of patients defined by genotype of the HTR2A SNP, rs7330461, race, and ethnicity, were evaluated as a part of the primary objective. LY2140023 did not separate from placebo in the primary efficacy endpoint (i.e., change in PANSS total score) in either the overall or predefined subpopulation at the 2 doses investigated (40 mg and 80 mg BID). The active control, risperidone, did separate from placebo in both populations. Therefore, HBBM is a negative study.

In an earlier clinical trial (Study HBBI), none of the LY2140023 dose arms (5 mg, 20 mg, 40 mg, 80 mg BID), nor the active control, olanzapine (15 mg), separated from placebo in the treatment of acute schizophrenia symptoms likely due to a higher than expected placebo response (−14.6 points) [7]. Research has highlighted an increasing placebo response in schizophrenia clinical trials [17], and enrichment designs have been developed to separate early placebo responders from randomized patients [18]. This study utilized several methods to attempt to prevent an abnormal placebo response, including the use of blinded independent raters and a 1-week placebo lead-in phase to identify “placebo responders” who were excluded from the primary efficacy analysis. The resulting mean PANSS total response of patients randomized to placebo in HBBM was not found to be excessive and was consistent with similar conclusive antipsychotic drug efficacy clinical trials reported in the literature [19,20]. However, despite the utilization of those methods to improve signal detection, LY2140023 did not show greater improvement compared to placebo. Furthermore, sensitivity analyses that included the placebo responders did not significantly alter treatment outcome. Therefore, the trial was conclusive in not showing antipsychotic efficacy for LY2140023 in the populations studied. In addition, another clinical trial (Study HBBN) with a similar study design and 10-mg, 40-mg and 80-mg BID doses was stopped for futility based upon an interim analysis (early termination; NCT01307800).

The time to all-cause discontinuation was shorter on both doses of LY2140023 than on placebo. While time to discontinuation due to AEs was comparable between the LY2140023 and placebo treatment groups, time to discontinuation due to lack of efficacy was shorter on both doses on LY2140023 than on placebo. The reasons why patients discontinued sooner on either dose of LY2140023 compared to placebo are unclear, although “Perceived Lack of Efficacy-Physician Decision” reported as a reason for early study discontinuation differed significantly among groups (LY2140023 80-mg BID group > placebo). Patients in the risperidone group had a significantly longer time to discontinuation due to lack of efficacy compared to those treated with placebo.

Both LY2140023 doses were found to be generally safe and well tolerated. The most common TEAEs were headache, schizophrenia-related events, nausea, insomnia, and increased blood CK, and these observed TEAEs are generally consistent with those reported in previous trials [7,21]. The TEAEs seen in patients treated with risperidone were consistent with the known AE profile of this antipsychotic. While significant differences between treatment groups and placebo were observed in laboratory analytes and vital signs, those differences were generally small and not considered to be clinically relevant. Although the AE of extrapyramidal disorders was significantly associated with 80 mg LY2140023, this event was not reflected in rating scales. LY2140023 was not associated with weight changes compared to placebo.

Three patients experienced a seizure during the study, although none occurred in either LY2140023 dose group. The aggregate data as of June 2012 provides crude and exposure-adjusted incidence rates which are comparable to the rates of atypical antipsychotics [22]. The crude incidence rate was calculated to be 0.264% and an exposure adjusted incidence (per patient year) of 0.011 (Lilly data on file).

The increase in prolactin levels due to treatment with risperidone (+50.90 ug/L) was anticipated and is consistent with the reported literature. The statistically significant increase in mean serum prolactin with LY2140023 80 mg BID (+1.16 ug/L) has not been seen previously, although the magnitude of this increase is small and may reflect normal variance, and its significance relative to placebo may have been influenced by the decrease in mean prolactin with placebo treatment.

### Limitations

Selecting Visit 3 for baseline due to the blinded placebo lead-in design may have resulted in a baseline PANSS total score that does not fully represent the acute state of illness of patients at Visit 1. The placebo lead-in may have led to the discontinuation of otherwise appropriate patients to have studied in this trial and lessened the response to treatment on efficacy scales. The high discontinuation rate on LY2140023 represents a significant number of missing patients whose potential data could have informed the final results differently (eg, better understanding of who are the patients who cannot tolerate LY2140023).

## Conclusion

Treatment with LY2140023 at the studied doses was not efficacious in improving symptoms of an acute exacerbation of illness in patients suffering from schizophrenia as studied in this trial. Overall, LY2140023 was generally well tolerated with no new significant adverse safety findings compared to previous trials. Further understanding of the role of glutamate as a therapeutic target in schizophrenia is needed.

## Additional file

Additional file 1:
**Plasma LY2140023 and LY404039 concentrations versus time.**
