# Supplementary material for: A double-blind, placebo-controlled comparator study of LY2140023 monohydrate in patients with schizophrenia
Source: BMC Psychiatry. 2014 Dec 10;14:351. doi: 10.1186/s12888-014-0351-3 (PMC4276262; doi:10.1186/s12888-014-0351-3)
Supplement: Additional file 1: — Plasma LY2140023 and LY404039 concentrations versus time. [file 12888_2014_351_MOESM1_ESM.docx]

**HBBM Additional file 1 - Figure:**

Plasma LY2140023 and LY404039 concentrations versus time. Plasma samples for assessment of LY2140023 and LY404039 concentrations were obtained at Visits 4, 5, and 9. The samples were analyzed for LY2140023 and LY404039 using a validated liquid chromatography with tandem mass spectrometry (LC/MS/MS) method at Advion BioServices, Inc. (Ithaca, NY, USA). A total of 7758 samples (3879 LY2140023 and LY404039 concentrations) were collected from 543 patients. The dataset used for pharmacokinetics (PK) model development included 3357 LY2140023 observations (499 patients) and 3420 LY404039 observations (503 patients). A population PK model was developed in which parameters for both LY2140023 and LY404039 were determined simultaneously by including a one-compartment sub-model for each analyte in a combined parent-metabolite model. The population PK model adequately characterized LY2140023 and LY404039 PK as shown by the concordance of the model predicted concentrations with the observations collected during the study.

Figure. LY2140023 concentrations (top row) and LY404039 concentrations (bottom row) over time after 40 mg (left column) and 80 mg (right column) at Visits 4, 5, and 9. The circles are study observations. The line and shaded area represent the population PK model predicted median and 90^th^ prediction interval.
